# Circadian Transcriptomic Dynamics Identify Transferable Retina–Choroid Expression Patterns in Myopia Development via Multistage Machine Learning

**DOI:** 10.3390/biology15110849

**Published:** 2026-05-29

**Authors:** Akarapon Watcharapalakorn, Teera Poyomtip, Patarakorn Tawonkasiwattanakun, Putri Krishna Kumara Dewi, Thotsapol Thomrongsuwannakij, Tanakamol Mahawan

**Affiliations:** 1 Faculty of Optometry, Ramkhamhaeng University, Bangkok 10240, Thailand; akarapon.w@rumail.ru.ac.th (A.W.); teera.p@rumail.ru.ac.th (T.P.); patarakorn.t@rumail.ru.ac.th (P.T.); 2 Medical Biochemistry Division, Department of Biomedical Science, Faculty of Medicine, Universitas Pendidikan Ganesha, Singaraja 81116, Indonesia; kumaradewi@undiksha.ac.id; 3 Akkhraratchakumari Veterinary College, Walailak University, Nakhon Si Thammarat 80160, Thailand; thotsapol.th@wu.ac.th; 4 One Health Research Center, Walailak University, Nakhon Si Thammarat 80160, Thailand; 5 Research Center for Theoretical Simulation and Applied Research in Bioscience and Sensing, Walailak University, Nakhon Si Thammarat 80160, Thailand

**Keywords:** myopia, circadian rhythm, RNA-seq, machine learning, retina–choroid axis, gene expression, transcriptomics, form-deprivation myopia

## Abstract

Circadian regulation has emerged as an important modulator of ocular growth; however, its role in organizing retina–choroid transcriptomic responses during myopia development remains incompletely understood. In this study, we reanalyzed publicly available retinal and choroidal RNA-seq datasets from chick models of form-deprivation myopia using a multistage machine learning framework. A biologically motivated ZT8/12 circadian window was defined from prior published time-of-day transcriptomic evidence and evaluated using feature selection, cross-tissue and cross-stage validation, and external validation in an independent retinal dataset. Machine learning models classified the ZT8/12 window with high performance across onset and progression datasets, and control analyses indicated that this signal reflects a broad transcriptome-wide temporal state rather than a pattern unique to the 53-gene signature. The final gene signature is therefore interpreted as a stable representative subset of the ZT8/12-associated expression state. Cross-species functional enrichment and ortholog mapping suggested hypothesis-generating functional relationships between chicken genes and human orthologs. Overall, this work provides a computational framework for evaluating time-associated expression patterns in myopia and highlights circadian timing as a candidate component of retina–choroid biology requiring further functional validation.

## Simple Summary

Myopia, or short-sightedness, is becoming increasingly common worldwide and can increase the risk of serious eye disease later in life. Although light exposure and daily body rhythms are thought to influence eye growth, the biological link between time of day and myopia development remains unclear. In this study, we reanalyzed publicly available gene activity data from the retina and choroid, two eye tissues involved in controlling eye growth, using computer-based prediction methods. The study confirmed previous findings that samples collected during the middle to late light period show a distinct pattern of gene activity. This pattern was observed across eye tissues and stages of myopia development and was partly supported in an external transcriptomic dataset. These findings suggest that the timing of biological processes in the eye may be related to myopia development. The study provides a useful framework for future research on how daily rhythms affect eye growth and may help guide future studies on the timing of myopia prevention or treatment strategies.

## 1. Introduction

Myopia (nearsightedness) is a major global public health concern, with projections suggesting that nearly half of the world’s population will be affected by 2050 [1]. In addition to the burden of refractive error, myopia—particularly high myopia—is associated with an increased risk of sight-threatening complications, including retinal detachment, myopic maculopathy, glaucoma, and choroidal neovascularization [2,3]. Despite extensive research, the biological mechanisms underlying abnormal ocular elongation remain incompletely understood.

Refractive development is a visually guided process in which the retina detects optical defocus and initiates signaling cascades that propagate through the choroid to the sclera, ultimately regulating eye growth [4]. This retina–choroid–sclera signaling axis integrates visual input, neuromodulation, metabolic regulation, and extracellular matrix remodeling [5]. Notably, pathological changes are often first observed in the retina, supporting its central role in initiating growth-related responses [6].

Circadian biology has increasingly been recognized as an important regulator of ocular growth [7]. In chicks and mice, manipulating circadian timing or light–dark cycles alter ocular growth. Similarly, in humans, reduced daylight exposure and irregular sleep patterns increase the risk of myopia onset, suggesting the involvement of daytime light and circadian entrainment in myopia development [8,9]. However, the molecular mechanisms linking circadian regulation to refractive development remain poorly defined.

The chick (*Gallus gallus domesticus*) is a well-established model for visually induced myopia [10]. Form-deprivation myopia (FDM) can be reliably induced using a translucent diffuser, producing rapid axial elongation and retinal and choroidal changes that resemble human myopia. Importantly, chicks exhibit robust circadian rhythms in retinal physiology, making them suitable for investigating time-of-day-dependent mechanisms [11].

Recent transcriptomic studies have characterized diurnal gene expression in the chick retina and choroid during myopia onset and progression, revealing pronounced time-of-day-dependent differences between occluded and control eyes [12,13]. These published analyses reported particularly strong transcriptional activity during the mid-to-late light phase, including ZT8/12. In the present study, we treat this interval as a biologically motivated analytical window rather than as a window newly discovered from model performance. This distinction is important because the primary datasets used here are derived from the same published studies that first described the temporal transcriptomic patterns.

Machine learning (ML) approaches have been increasingly used in myopia research to identify molecular biomarkers and model complex gene expression patterns associated with ocular growth [14,15]. By capturing nonlinear and multivariate relationships in high-dimensional data, ML enables robust classification and cross-dataset validation of myopia-related molecular signatures, typically using differential expression and feature selection methods. While these approaches have identified candidate biomarkers, they are generally based on single datasets and do not incorporate temporal or circadian structure [16]. More recent models integrating clinical and metabolomic features have shown promising predictive performance, but they do not account for circadian dynamics [17]. Thus, a key gap remains in applying integrative, multi-stage ML frameworks to circadian transcriptomic data in myopia.

In this study, we integrate publicly available retinal and choroidal RNA-seq datasets using a multistage machine learning framework to evaluate whether the biologically motivated ZT8/12 interval can be represented by a stable and transferable multigene expression pattern. We assess feature-selection stability, cross-tissue and cross-stage transferability, external reproducibility, and control analyses designed to distinguish time-associated expression structure from arbitrary label assignment or non-specific transcriptomic variation. We further use cross-species functional enrichment as a hypothesis-generating analysis rather than as direct evidence of conserved causal mechanisms.

## 2. Materials and Methods

### 2.1. Data Collection

This study reanalyzed publicly available RNA-seq datasets from previously published chick myopia studies (Stone et al., 2024 [12,13]), including GSE227724 (myopia onset) and GSE261232 (myopia progression). Both datasets were generated from Cornell K strain White Leghorn chicks subjected to form-deprivation myopia under 12 h light/12 h dark conditions. Retina and choroid tissues were collected at six Zeitgeber time points (ZT0-ZT20, 4 h intervals; *n* = 6 chicks per time point). Count matrices and metadata were inspected before normalization. Count values of zero were treated as observed low or absent expression values rather than missing data and were not imputed. No missing expression values remained after preprocessing. Raw sequencing data and expression matrices were obtained from Gene Expression Omnibus and used for downstream analyses.

### 2.2. Circadian Critical Window Identification

The ZT8/12 classification target was defined as a biologically motivated analytical contrast based on previously reported time-of-day transcriptomic patterns in the source datasets, rather than as a de novo discovery derived from model performance in the present study. Because circadian transcriptomes are periodic, this binary grouping does not fully capture temporal continuity or phase relationships across the 24 h cycle. Instead, the aim was to test whether the previously reported mid-to-late light-phase signal could be represented as a transferable multigene expression signature across tissues, disease stages, and an external dataset.

Samples collected at ZT8 and ZT12 were grouped as ZT_8/12 to form the primary analytical class, while the remaining time points were grouped as ZT_other. This classification framework should therefore be interpreted as a targeted validation of a biologically motivated time window, rather than as a comprehensive rhythmicity model. Dedicated rhythmicity models will be required in future work to estimate phase, amplitude, and temporal autocorrelation more directly.

### 2.3. Machine Learning Analysis

**Figure 1:**
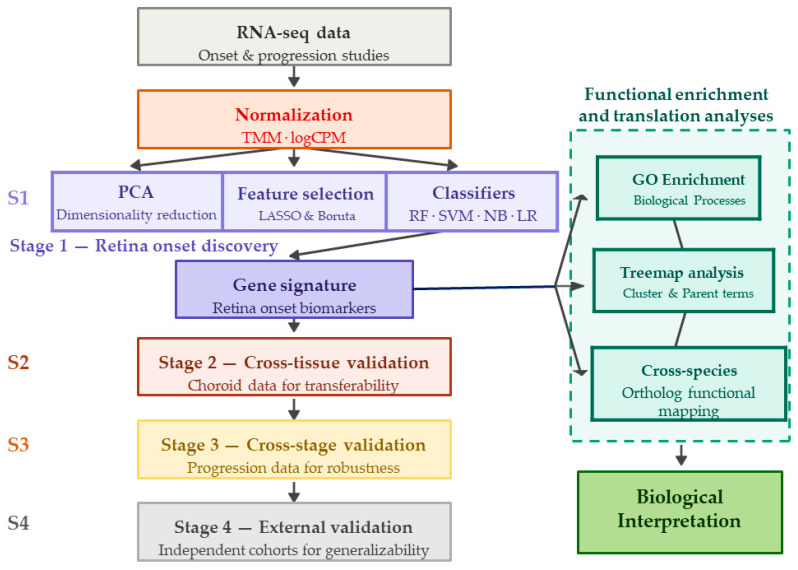
Multistage machine learning framework for evaluating time-associated expression patterns in myopia development. RNA-seq data from onset and progression studies were normalized and analyzed through a four-stage pipeline. Stage 1 (S1) to Stage 4 (S4). Downstream functional analyses provide insights into circadian regulation and retina–choroid interactions underlying myopia development.

The machine learning framework consisted of four stages (Figure 1): (i) feature discovery in the retina onset dataset, (ii) cross-tissue validation in the choroid onset dataset, (iii) cross-stage validation using the progression dataset, and (iv) external validation using an independent retinal dataset. All analyses were conducted in R version 4.5.1 [18]. The complete analysis scripts, processed metadata, model outputs, and R session information are archived on Zenodo (https://doi.org/10.5281/zenodo.19953551; accessed on 1 May 2026). Key packages included edgeR v4.6.3 [19], Boruta v9.0.0 [20], caret v7.0.1 [21], clusterProfiler v4.16.0 [22], rrvgo v1.20.0 [23], alluvial v12.5.1 [24], and iml v0.11.4 [25]. Model training and validation were performed on a Windows 11 workstation (Microsoft Corporation, Redmond, WA, USA) with a 13th Gen Intel(R) Core (TM) i5-13420H CPU and 16 GB RAM (Intel Corporation, Santa Clara, CA, USA). Random seeds were fixed for all resampling, feature-selection, and control-analysis steps.

This study follows the TRIPOD + AI (Transparent Reporting of a Multivariable Prediction Model for Individual Prognosis or Diagnosis—Artificial Intelligence extension) guidelines [26], ensuring transparent and reproducible reporting of the machine learning models used for predictive analysis in myopia. The TRIPOD + AI checklist, outlining the study’s adherence to these guidelines, can be found in Supplementary Data S6.

#### 2.3.1. Stage 1: Primary Discovery Model (Retina—Onset Dataset)

RNA-seq count matrices and sample metadata were retrieved from GSE227724. Raw counts were processed using edgeR v4.6.3 [19]. Low-expression genes were filtered using *filterByExpr* function, library sizes were normalized using the Trimmed Mean of M-values (TMM) method [27], and expression values were transformed to log2 counts per million (logCPM). Zeitgeber time was encoded as sine and cosine variables for exploratory circadian visualization and metadata-aware description of sampling. To avoid target leakage, these time-encoding variables were not included in Boruta feature selection, LASSO feature selection, or supervised classifier training. All predictive models were trained using gene-expression features only.

To avoid data leakage, feature selection and model development were restricted to the training partitions. Feature selection was performed using Boruta v9.0.0 [20]. Boruta was repeated 50 times using different random seeds, with maxRuns = 100 for each run. Only genes selected in all 50 runs were retained as the final consensus signature. LASSO was used as a parsimonious linear comparator, whereas Boruta was used as the primary downstream signature because it is an all-relevant selection method capable of retaining correlated and potentially interacting genes.

Classification models were developed using Random Forest (RF), Support Vector Machine (SVM), Naive Bayes (NB), and regularized Logistic Regression (LR) within caret v7.0.1 [21]. These algorithms were selected to represent complementary model assumptions: RF as a nonlinear ensemble method, SVM as a margin-based classifier for high-dimensional data, NB as a low-complexity probabilistic baseline, and LR as an interpretable linear benchmark with regularization. Deep learning models were not used because the sample size was modest, and the risk of overfitting was high. Feature-selection stability was assessed using Boruta selection-frequency plots, and SHAP analysis was performed using the final RF classifier trained on the 53-gene signature by iml v0.11.4 [25]. Mean absolute SHAP values were used to summarize global feature importance, and SHAP summary plots were generated for the top contributing genes.

Importantly, explicit oversampling techniques (e.g., SMOTE) were not used to artificially balance the classes before training. Instead, the models were evaluated for their ability to handle the natural class distributions, with performance metrics calculated on the 20% hold-out test sets. These metrics included Accuracy, Precision, Recall, F1-score, Area Under the Curve (AUC), and Matthew’s correlation coefficient (MCC). Recall, F1-score, and MCC were prioritized to assess the models’ ability to classify the data accurately in the presence of class imbalance, without relying on synthetic data augmentation. Summary statistics and 95% confidence intervals were calculated for all metrics across the 50 outer iterations. Gene signatures derived from this retina onset dataset were then evaluated for cross-tissue and cross-stage transferability.

#### 2.3.2. Stage 2: Cross-Tissue Validation (Retina → Choroid)

To evaluate transferability across tissues, the gene signatures derived from the retina onset dataset were applied to choroid samples from the same study. Models were trained using the same nested cross-validation framework, incorporating 5-fold inner cross-validation for hyperparameter tuning and 50 outer iterations of stratified resampling. The same four algorithms (RF, SVM, NB, LR) were employed. Performance was assessed using the same stringent evaluation metrics (Accuracy, Precision, Recall, F1-score, AUC, MCC) to ensure robustness across tissues.

#### 2.3.3. Stage 3: Cross-Stage Validation (Onset → Progression)

To assess temporal robustness, the retina-derived gene signatures were applied to the independent progression dataset. Models were trained using the same algorithms and circadian classification criteria (ZT_8/12 vs. ZT_other). Classification was performed using the established 50-iteration nested cross-validation procedure. Model performance was subsequently quantified using the previously defined metrics, with particular emphasis on Recall, F1-score, and MCC, to determine whether the chronobiological signatures identified during the onset phase remained predictive and resilient to class imbalance during disease progression.

#### 2.3.4. Stage 4: External Validation (Independent Dataset)

External validation was performed using the independent retinal RNA-seq dataset GSE203604 (*n* = 42). Raw count data were processed in R, duplicate gene identifiers were collapsed by averaging, and lowly expressed genes were filtered using a threshold of counts ≥10 in at least three samples. Expression values were log2-transformed, and the predefined 53-gene Boruta signature was intersected with genes detected in the external dataset, resulting in 32 available genes. Genes absent from the external dataset were excluded rather than imputed. Gene-wise z-score normalization was applied before downstream analysis. Because GSE203604 differs from the primary form-deprivation datasets and does not include the same six Zeitgeber sampling points, it was used to evaluate whether the predefined signature retained multivariate structure in an independent retinal myopia dataset rather than to repeat the ZT8/12 classification directly. Classification performance was evaluated using nested leave-one-out cross-validation with regularized Logistic Regression and a linear SVM. Statistical significance was assessed using permutation testing, and 95% confidence intervals were estimated by bootstrap resampling. A composite signature score was calculated as the mean gene-wise z-score across detected signature genes, and temporal trends across D1, D3, and D6 were evaluated using linear models including day, group, and interaction terms.

#### 2.3.5. Hyperparameter Tuning

For Stages 1–3, model performance was evaluated using repeated nested resampling. The outer loop consisted of 50 iterations of stratified resampling, in which data were split into 80% training and 20% testing partitions while preserving the ZT8/12 and ZT_other class distributions. Within each training partition, 5-fold inner cross-validation was used to select optimal hyperparameters based on AUC. Reported performance values represent the mean and 95% confidence interval across the 50 outer test-set evaluations. For Stage 4 external validation, AUC confidence intervals were estimated by bootstrap resampling.

For RF, *ntree* was set to 500, and *mtry* was tuned within the training folds. For Stages 1–3, SVM models were implemented using the radial-basis kernel through the caret svmRadial method, with centering and scaling applied within training folds. Naive Bayes was tuned using Laplace smoothing parameters, and regularized Logistic Regression was tuned across alpha and lambda values. For Stage 4 external validation, a linear SVM was used because the dataset was smaller and only 32 signature genes were available after gene-overlap filtering.

#### 2.3.6. Control and Sensitivity Analyses

To evaluate whether classification performance depended on the ZT8/12-associated expression structure rather than arbitrary label assignment or non-specific transcriptomic variation, several control and sensitivity analyses were performed. First, a time-label permutation control was conducted by randomly shuffling the ZT8/12 and ZT_other class labels while preserving the expression matrix and the 53-gene feature set. Second, random 53-gene sets were sampled from the expression matrix and evaluated using the same Random Forest workflow. Third, six candidate housekeeping/reference genes that overlapped with the dataset were examined for Zeitgeber-time association and tested as a reference-gene control. Fourth, data-derived non-circadian genes were selected from genes with weak Zeitgeber-time association and used to generate matched 53-gene control sets. Finally, single-time-point sensitivity analyses were performed for ZT8 alone and ZT12 alone and compared with the combined ZT8/12 window.

### 2.4. Functional Enrichment and Translation Analyses

To investigate biological processes associated with the selected gene signature, Gene Ontology (GO) enrichment analysis was performed on Boruta-selected chicken genes and their corresponding human orthologs using clusterProfiler v4.16.0 [22]. Significant GO terms (adjusted *p* ≤ 0.05) were summarized and reduced for redundancy using rrvgo v1.20.0 [23].

Parent GO terms for chicken and human datasets were visualized using treemaps. Enrichment results were also summarized at the parent biological-process level and visualized using alluvial diagrams generated with the alluvial package v12.5.1 [24]. These cross-species enrichment analyses were interpreted as hypothesis-generating because GO enrichment and ortholog mapping provide indirect functional evidence and may be affected by annotation bias and incomplete cross-species translatability.

## 3. Results

### 3.1. Study Design and Sample Size

The myopia onset and progression studies used identical circadian sampling schemes. Each study included 36 chicks in total, corresponding to six Zeitgeber time points with six chicks per time point. For each chick, tissues were collected from the occluded eye and the contralateral open eye, and retina and choroid were processed separately. Thus, each study contributed 144 tissue-level samples in total: 36 chicks × 2 eyes × 2 tissues. For tissue-specific analyses, retina and choroid were analyzed separately, corresponding to 72 samples per tissue per study. At the full tissue-level dataset scale, the ZT8/12 class included 48 samples, while the ZT_other class included 96 samples. Within each tissue-specific dataset, the ZT8/12 and ZT_other classes included 24 and 48 samples, respectively, as shown in Table 1. The paired origin of occluded and contralateral eyes is considered when interpreting model transferability.

### 3.2. ZT8/12 Analytical Window and Transcriptomic Structure

Samples collected at ZT8 and ZT12 were grouped as the ZT_8/12 analytical window, comprising 48 tissue-level samples (33.3%) in each study. The remaining time points (ZT0, ZT4, ZT16, and ZT20) were classified as ZT_other, representing 96 samples (66.7%), and were used as the comparison class for downstream machine learning analyses. Detailed phenotype metadata, including the computational class label, are provided in Supplementary Data S1.

**Figure 2:**
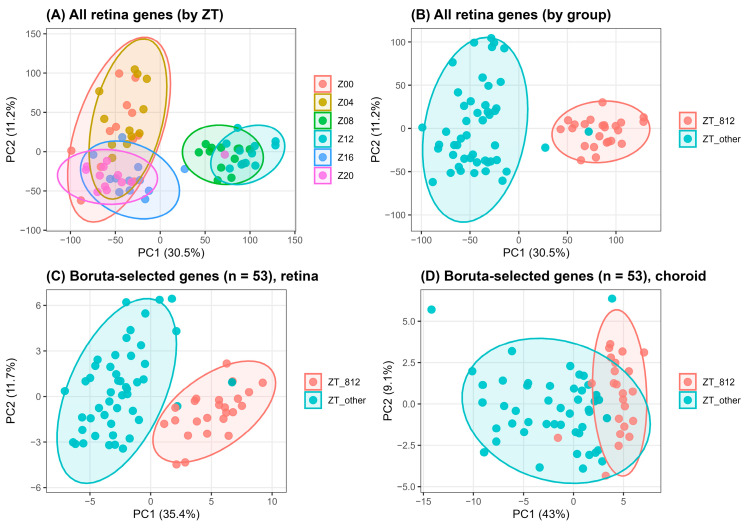
PCA of retinal and choroidal transcriptomic profiles. (**A**) Full retinal transcriptome (14,484 genes) by ZT point. (**B**) Full retina grouped by target window (ZT8/12 vs. others). (**C**) Retinal 53-gene Boruta signature. (**D**) Choroidal validation of the 53-gene signature. Axis labels indicate the percentage of variance explained by each principal component. Confidence ellipses indicate group-level dispersion and are shown for visualization only; they were not used for classifier training.

PCA showed that samples collected at ZT8/12 were separated from other time points, supporting the presence of a strong time-associated transcriptomic structure (Figure 2). This separation is interpreted as evidence of a broad ZT8/12-associated temporal state, not as proof that the 53-gene signature is unique or causal. Figure 2 includes the percentage of variance explained by each principal component, and confidence ellipses are shown for visualization only.

### 3.3. Machine Learning Classification and Control Analyses (Stage 1)

Feature selection was performed using the retina onset dataset. After repeated analysis, LASSO selected 24 genes, while Boruta identified 53 genes that were selected consistently in all 50 repeated runs (50/50). The 53 gene IDs and their ortholog annotations are provided in Supplementary Data S2. The 50/50 Boruta criterion supports feature-selection stability, although subsequent control analyses indicate that the ZT8/12 signal is broad across the transcriptome rather than restricted to these genes alone.

Supervised models trained on the retina onset dataset distinguished samples collected during the ZT8/12 window from other time points. Using the LASSO-selected gene set (*n* = 24), all classifiers showed high performance. Using the Boruta-selected gene set (*n* = 53), performance was also high across classifiers, supporting the utility of this stable consensus signature for downstream transferability testing. We therefore used the Boruta-selected genes for Stages 2–4, while retaining LASSO as a parsimonious comparator (Table 2).

Boruta feature-selection stability analysis showed that the final 53-gene signature consisted only of genes selected in all 50 repeated Boruta runs, supporting high selection consistency (Supplementary Figure S1). SHAP analysis of the final RF model identified the top contributing genes for ZT8/12 classification based on mean absolute SHAP values (Supplementary Figure S2). The SHAP summary plot further showed the direction and distribution of gene-level contributions across samples (Supplementary Figure S3). These analyses support model stability and interpretability but do not imply causal roles for individual genes.

To improve biological interpretation of the model features, the top SHAP-ranked genes were examined in relation to known retinal and circadian pathways. Several top SHAP-ranked genes were related to downstream retinal signaling, ion transport, lipid metabolism, and photoreceptor-associated processes, for example, *CACNA2D4*, *CNGA3*, *KCNJ11*, *FADS2*, *DGAT2*, *CERS2*, and *ESRRB*. Thus, the SHAP results suggest that the ZT8/12-associated signature reflects downstream retinal physiological and metabolic states rather than direct selection of canonical clock genes. These genes were further explained by GO enrichment visualized by Treemap (rrvgo v1.20.0 [23]) and alluvial visualizations(alluvial v12.5.1 [24]) in Section 3.7.

### 3.4. Control and Sensitivity Analyses for ZT8/12 Classification

Control analyses supported that the ZT8/12 classification depended on the true time-associated expression structure. In the time-label permutation analysis, the true-label RF model achieved an AUC of 1.000, whereas shuffled-label controls produced a mean AUC of 0.637 ± 0.034. None of the 100 shuffled-label permutations reached the observed AUC, corresponding to a corrected empirical *p*-value of 0.0099.

Random 53-gene sets also achieved high classification performance, with a mean AUC of 0.997 ± 0.003. This indicates that the ZT8/12 contrast reflects a broad transcriptome-wide temporal state rather than a signal unique to the 53 Boruta-selected genes. In contrast, data-derived non-circadian 53-gene controls showed lower and more variable performance, with a mean AUC of 0.667 ± 0.080 and mean MCC of 0.048 ± 0.256, supporting that classification depended on time-associated expression structure rather than arbitrary non-circadian gene sets.

Six candidate housekeeping/reference genes overlapped with the expression matrix. However, five of these six genes showed significant Zeitgeber-time association after false-discovery-rate correction, indicating that they were not suitable stable negative controls in this circadian retinal dataset. Single-time-point sensitivity analyses showed that ZT8-only and ZT12-only contrasts were both highly separable, while the combined ZT8/12 window showed the most stable performance across metrics. Full control and sensitivity results are provided in Supplementary Data S7.

### 3.5. Cross-Dataset and Cross-Tissue Validation (Stage 2 and 3)

To assess transferability of the ZT8/12-associated expression pattern, models trained on the retina onset dataset were evaluated in two validation settings: cross-stage validation using the progression dataset and cross-tissue validation using choroidal samples. In the cross-stage setting (onset to progression), SVM achieved the highest performance (accuracy = 0.972; AUC = 0.979), followed by RF (accuracy = 0.931; AUC = 0.896). In the cross-tissue setting (onset retina to choroid), RF showed strong performance (accuracy = 0.954; AUC = 0.946). These results support predictive transferability under matched experimental conditions, but they do not establish direct signaling transmission or causality between retina and choroid (Table 3).

### 3.6. External Validation on Independent Data (Stage 4)

**Figure 3:**
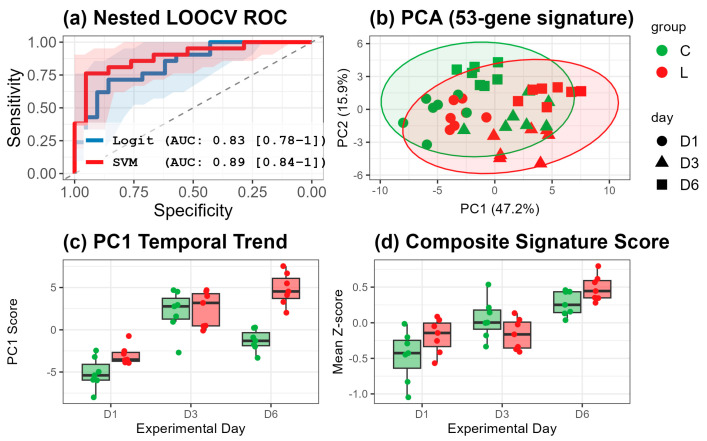
External validation of the 53-gene signature in GSE203604. (**a**) Receiver operating characteristic curves from nested leave-one-out cross-validation for regularized Logistic Regression and linear SVM models. AUC values and 95% bootstrap confidence intervals are shown. (**b**) PCA based on the 32 detected genes from the predefined 53-gene signature, showing sample distribution by experimental group and time point. (**c**) Temporal trend of PC1 scores derived from signature-gene expression. (**d**) Temporal trend of the composite signature score, calculated as the mean gene-wise z-score across detected signature genes. Error bars indicate standard error.

Of the 53 genes in the original Boruta signature, 32 were detected in GSE203604 and used for external validation. Unsupervised analyses based on these genes showed consistent multivariate structure, with PCA indicating separation of samples across time points and experimental groups. Nested leave-one-out cross-validation showed an AUC of 0.823 for regularized Logistic Regression and 0.912 for the linear SVM. Permutation testing indicated that the linear SVM performed significantly better than chance (*p* = 0.015), whereas Logistic Regression did not exceed chance-level performance (*p* = 0.68). Bootstrap confidence intervals were 0.782–0.997 for Logistic Regression and 0.841–0.998 for the linear SVM. The composite signature score increased from D1 to D6, and PCA showed variation along PC1 with differences between control and myopia groups across time as shown in Figure 3.

### 3.7. Cross-Species Functional Enrichment of Myopia-Associated Gene Signatures

**Figure 4:**
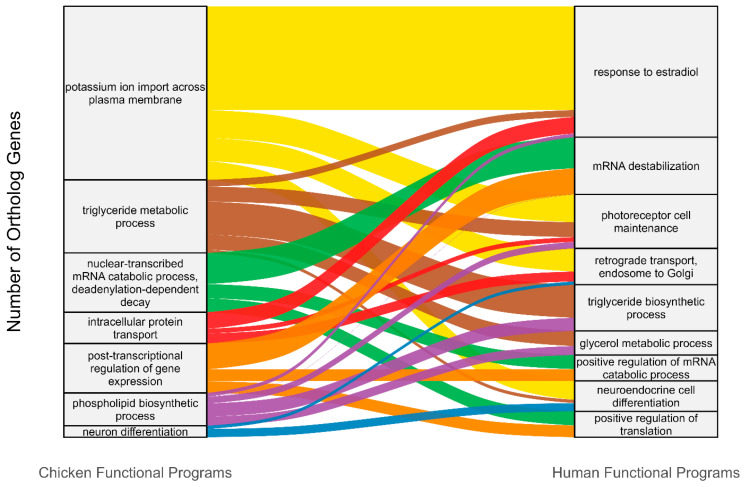
Functional Translation of Myopia Gene Programs. Major cellular programs in chicken are mapped to regulatory pathways in humans.

GO enrichment analysis of Boruta-selected genes revealed distinct but related functional profiles between chicken genes and their human orthologs. After redundancy reduction, enriched biological processes in chicken were summarized into parent programs associated with cellular metabolism, ion transport, intracellular trafficking, and post-transcriptional regulation (Supplementary Data S3). Human orthologs were enriched for categories related to neuroendocrine regulation, photoreceptor maintenance, hormonal response, and mRNA stability (Supplementary Data S4). Treemap and alluvial visualizations further highlighted these differences in enriched functional categories between chicken genes and their human orthologs (Figure 4; Supplementary Data S5).

These GO patterns provide mechanistic context for the SHAP-ranked genes and suggest that the Boruta-selected chicken genes and their human orthologs map to partially overlapping but distinct biological-process groups. The enrichment patterns are consistent with the gene-level interpretation that the ZT8/12-associated signature captures retinal signaling, photoreceptor-related function, ion transport, and metabolic regulation. However, these findings should be interpreted as functional annotation trends rather than direct evidence of conserved causal mechanisms, because GO enrichment and ortholog mapping provide indirect evidence and may be influenced by annotation bias, incomplete cross-species translatability, and the fact that enrichment does not establish causality or pathway direction.

## 4. Discussion

This study evaluated whether a biologically motivated ZT8/12 window, previously highlighted in published chick myopia transcriptomic studies, can be represented by a stable and transferable multigene expression pattern [12,13]. Using a multistage machine learning pipeline that produced high classification performance and cross-dataset transferability, our results indicate robust confirmatory evidence for the presence of a strong ZT8/12-associated transcriptomic state across onset and progression datasets. However, the classification framework should not be interpreted as a de novo discovery of the window or as a complete periodic time-series model. Rather, it provides a targeted test of a previously reported temporal interval. The ZT8/12 interval is biologically plausible in the context of ocular circadian physiology because it spans the mid-to-late light phase, a period associated with dynamic changes in retinal neuromodulation and transition toward the dark phase [28,29]. Nevertheless, the present computational analysis does not establish that this interval is mechanistically causal in myopia progression. It should instead be considered a hypothesis-generating temporal window for future rhythmicity-based and functional studies.

The control analyses refine the interpretation of the 53-gene signature. Although all 53 genes were selected consistently in 50 out of 50 repeated Boruta runs, random 53-gene controls also achieved high classification performance. This suggests that the ZT8/12 contrast reflects a broad transcriptome-wide temporal state rather than a signal uniquely restricted to the selected genes. Therefore, the 53-gene Boruta signature should be interpreted as a stable and compact representative subset of the ZT8/12-associated expression pattern, not as an exclusive or causal gene set. The lower performance of data-derived non-circadian gene controls further supports that the classifier depends on time-associated expression structure [30,31].

Cross-tissue validation demonstrated that a retinally derived expression signature could classify choroidal samples under matched experimental conditions. This finding supports cross-tissue predictive transferability, but it does not establish direct signaling transmission or causality between retina and choroid. Because retina and choroid samples were collected from the same animals and time points, shared timing and paired biological context may contribute to the observed performance [12,13]. Independent tissue-specific datasets and functional experiments will be required to confirm whether these transcriptomic patterns reflect coordinated retina–choroid signaling [28,32].

Cross-species enrichment analysis suggested that chicken signature genes and their human orthologs are associated with partially overlapping but distinct functional categories. Human orthologs were enriched for regulatory pathways related to neuroendocrine signaling, photoreceptor maintenance, hormonal response, and mRNA stability, a pattern broadly consistent with GWAS and functional genomic studies implicating photoreceptor-related pathways, synaptic or neuroregulatory signaling, and post-transcriptional regulation in myopia development [33,34,35]. In contrast, chicken genes were more strongly associated with cellular metabolic and regulatory processes [36]. These findings suggest partial translational convergence at the pathway level, while also indicating species-specific differences in downstream biological implementation. Such differences may reflect biological divergence and species-specific regulatory context, but they may also result from ortholog-mapping limitations, gene-annotation bias, or incomplete translatability of the chick model to human myopia [37]. Therefore, this cross-species analysis should be considered hypothesis-generating rather than direct evidence of neuroendocrine reorganization in humans. Nevertheless, the presence of partially shared functional themes supports the continued relevance of the chick model for studying fundamental mechanisms of visually induced myopia [11].

The absence of canonical core clock genes such as *CLOCK* and *BMAL1*/*ARNTL* among the top SHAP-ranked genes suggests that the classifier captured downstream circadian-associated retinal states rather than the central molecular clock itself. This is biologically plausible because retinal circadian clocks regulate multiple downstream processes, including gene expression, synaptic communication, neurotransmitter release, metabolism, and photoreceptor physiology [38]. In the context of myopia, the mid-to-late light phase may be particularly relevant because retinal dopamine is generally associated with light-adapted retinal signaling and inhibition of excessive ocular elongation, whereas melatonin-related pathways are more closely linked to the transition toward the dark phase [39,40]. The presence of SHAP-ranked genes related to photoreceptor signaling, including *CACNA2D4* and *CNGA3*, ion transport through *KCNJ11*, lipid metabolism through *FADS2*, *DGAT2*, and *CERS2*, and photoreceptor maintenance through ESRRB supports the interpretation that the ZT8/12 signature may reflect a coordinated downstream retinal signaling and metabolic state [41,42,43]. Nevertheless, these associations remain correlative and require functional validation.

External validation provided partial support that the predefined 53-gene signature retains reproducible multivariate structure in an independent retinal RNA-seq dataset. Although regularized Logistic Regression showed moderate classification performance, it did not exceed chance-level performance based on permutation testing. In contrast, the linear SVM achieved higher and statistically significant performance, suggesting that the detected signature genes capture multivariate expression patterns that are partly preserved outside the primary datasets [44].These findings should be interpreted cautiously because GSE203604 had a modest sample size, differed in experimental design, lacked matched circadian sampling, and contained only 32 of the 53 original signature genes.

A further limitation is that the main modelling strategy used a binary ZT8/12 versus other-time-point classification task. This approach was useful for testing a specific biologically motivated time window, but it does not fully capture the periodic and continuous nature of circadian transcriptomic data. Future studies should incorporate dedicated rhythmicity or longitudinal models, such as harmonic regression [45], cosinor-based modelling [46], or mixed-effects time-series approaches, to estimate phase, amplitude, and temporal autocorrelation more directly.

Several additional limitations should be acknowledged. This study relies on publicly available RNA-seq datasets with modest sample sizes, reflecting the constraints of circadian experimental designs requiring terminal tissue collection. The chick model is well established for visually induced myopia, but species-specific differences may limit direct translation to human disease [11]. The analysis was restricted to transcriptomic data, and other regulatory layers, including proteomic and metabolic processes, were not assessed. Functional validation experiments were not performed. Finally, paired-eye and paired-tissue origins may contribute to some cross-dataset transferability patterns and should be addressed in future experimental designs [47].

These findings identify circadian timing and retina–choroid expression patterns as candidate mechanisms requiring functional validation. Because this study did not test interventions, the results should not be interpreted as direct evidence that manipulating circadian timing will alter myopia progression. Instead, the ZT8/12 window provides a hypothesis-generating temporal framework for future mechanistic, rhythmicity-based, and chronotherapeutic studies [48,49,50].

## 5. Conclusions

This study shows that a biologically motivated ZT8/12 window can be represented by a transferable multigene expression pattern in chick retinal and choroidal transcriptomic datasets. The findings support cross-stage and cross-tissue predictive transferability of this time-associated expression state, with partial support from an independent retinal dataset. However, the results do not establish causality or direct retina–choroid signaling mechanisms. The 53-gene Boruta signature should be interpreted as a stable representative subset of a broader ZT8/12-associated transcriptomic state, not as an exclusive or causal gene set. These findings provide a computational framework and candidate gene set for future functional and rhythmicity-based studies of circadian regulation in myopia development.

## Figures and Tables

**Table 1:** Experimental design, sampling scheme, and circadian classification.

Study	Duration	Day	Rep/ZT	Total Chicks	Samples per Tissue	Total Tissue-Level Samples	Class	*n* (%)	n per Tissue
Onset	1 cycle	1	6	36	72	144	ZT_8/12	48 (33.3)	24
							ZT_other	96 (66.7)	48
Progression	4 cycles	5	6	36	72	144	ZT_8/12	48 (33.3)	24
							ZT_other	96 (66.7)	48

Note: Total tissue-level samples were calculated as 36 chicks × 2 eyes × 2 tissues = 144 samples per study. Tissue-specific analyses were conducted separately for retina and choroid, resulting in 72 samples per tissue per study. ZT8/12 included samples from ZT8/12, whereas ZT_other included ZT0, ZT4, ZT16, and ZT20.

**Table 2:** Performance of machine learning classifiers for identifying the ZT8/12 analytical window in the retina onset dataset. Values are mean ± 95% confidence interval across 50 outer resampling iterations.

Metric	RF	SVM	NB	LR
LASSO (*n* = 24)				
Accuracy	0.909 ± 0.081	0.920 ± 0.077	0.831 ± 0.106	0.943 ± 0.066
Precision	0.946 ± 0.064	0.951 ± 0.061	0.967 ± 0.051	0.949 ± 0.062
Recall	0.924 ± 0.075	0.938 ± 0.068	0.789 ± 0.115	0.973 ± 0.046
F1-Score	0.933 ± 0.071	0.940 ± 0.067	0.860 ± 0.098	0.960 ± 0.055
AUC	0.952 ± 0.060	0.950 ± 0.062	0.957 ± 0.057	0.969 ± 0.049
MCC	0.802 ± 0.113	0.828 ± 0.107	0.684 ± 0.131	0.868 ± 0.096
BORUTA (*n* = 53)				
Accuracy	0.957 ± 0.057	0.932 ± 0.071	0.888 ± 0.089	0.938 ± 0.068
Precision	0.971 ± 0.047	0.971 ± 0.048	0.965 ± 0.052	0.944 ± 0.065
Recall	0.969 ± 0.049	0.933 ± 0.071	0.873 ± 0.094	0.973 ± 0.046
F1-Score	0.969 ± 0.049	0.948 ± 0.063	0.911 ± 0.080	0.957 ± 0.057
AUC	0.964 ± 0.053	0.950 ± 0.062	0.957 ± 0.058	0.958 ± 0.057
MCC	0.905 ± 0.083	0.860 ± 0.098	0.776 ± 0.118	0.858 ± 0.099

Abbreviations: RF, Random Forest; SVM, Support Vector Machine; NB, Naïve Bayes; LR, Logistic Regression.

**Table 3:** Cross-stage and cross-tissue validation performance of machine learning classifiers.

Metric	Cross-Stage Validation (Onset → Progression)				Cross-Tissue Validation (Onset: Retina → Choroid)			
	RF	SVM	NB	LR	RF	SVM	NB	LR
Accuracy	0.931	0.972	0.847	0.403	0.954	0.898	0.826	0.618
Precision	1.000	0.923	1.000	0.358	0.937	0.846	0.696	0.428
Recall	0.792	1.000	0.542	1.000	0.925	0.855	0.925	0.615
F1	0.884	0.960	0.703	0.527	0.925	0.837	0.774	0.512
AUC	0.896	0.979	0.771	0.448	0.946	0.886	0.827	0.630
MCC	0.847	0.941	0.664	0.193	0.898	0.777	0.684	0.226

Abbreviations: RF, Random Forest; SVM, Support Vector Machine; NB, Naïve Bayes; LR, Logistic Regression.

## Data Availability

Publicly available GEO datasets GSE227724, GSE261232, and GSE203604 were used in this study. Additional supporting data and results are provided in the Supplementary Materials. The machine learning framework and R scripts used for analysis (including Stages 1 through 4) are archived and publicly available on Zenodo at https://doi.org/10.5281/zenodo.19953551 (accessed on 1 May 2026).

## Supplementary Materials

The following supporting information can be downloaded at: https://www.mdpi.com/article/10.3390/biology15110849/s1. Supplementary Data S1: Phenotypic metadata and ZT8/12 class labels. Supplementary Data S2: Boruta-selected genes, ortholog annotations, and feature information. Supplementary Data S3: Gene Ontology enrichment results for chicken genes. Supplementary Data S4: Gene Ontology enrichment results for human orthologs. Supplementary Data S5: Treemap and alluvial visualization data for functional enrichment results. Supplementary Data S6: TRIPOD + AI checklist [26,51]. Supplementary Data S7: Control and sensitivity analyses for ZT8/12 classification. Supplementary Figure S1: Boruta feature-selection frequency across 50 repeated runs. Supplementary Figure S2: SHAP global feature importance for the top 20 genes. Supplementary Figure S3: SHAP summary plot for the top 20 Boruta genes.

## Abbreviations

The following abbreviations are used in this manuscript:

RF	Random Forest
SVM	Support Vector Machine
NB	Naïve Bayes
LR	Logistic Regression
AUC	Area Under the Curve
MCC	Matthew’s Correlation Coefficient
PCA	Principal Component Analysis
GO	Gene Ontology
TMM	Trimmed Mean of M-values
