# Supplementary material for: Circadian Transcriptomic Dynamics Identify Transferable Retina–Choroid Expression Patterns in Myopia Development via Multistage Machine Learning
Source: Biology (Basel). 2026 May 29;15(11):849. doi: 10.3390/biology15110849 (PMC13256023; doi:10.3390/biology15110849)

Treemap Analysis

Gene Ontology (GO) Biological Process enrichment was performed for chicken gene IDs and their corresponding human orthologs. Full results are provided in Supplementary Data S3 and S4. Venn diagram analysis showed that 76 of 136 significant GO terms identified in chicken overlapped with those in humans, while these 76 terms represent a smaller proportion of the 697 significant GO terms identified in humans. This indicates the presence of conserved biological processes across species, alongside a broader and more complex functional landscape in humans, as further illustrated by the treemap analysis.

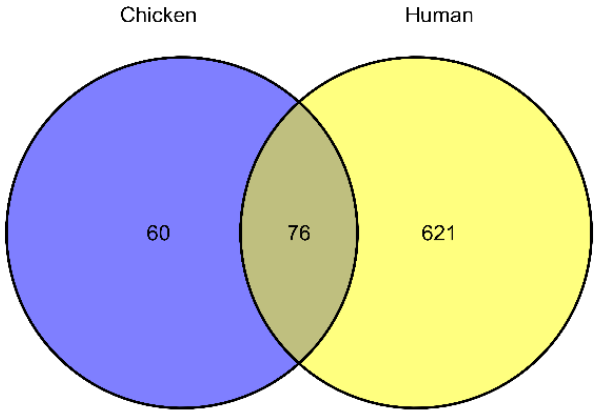

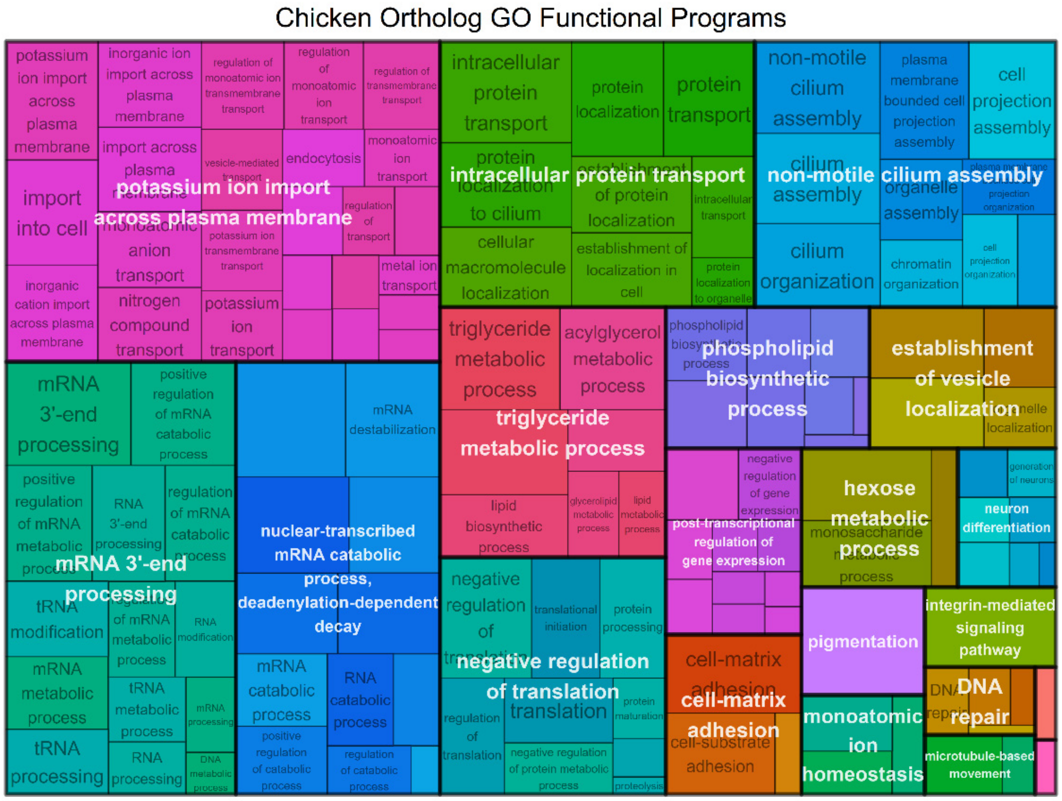

Human Ortholog GO Functional Programs

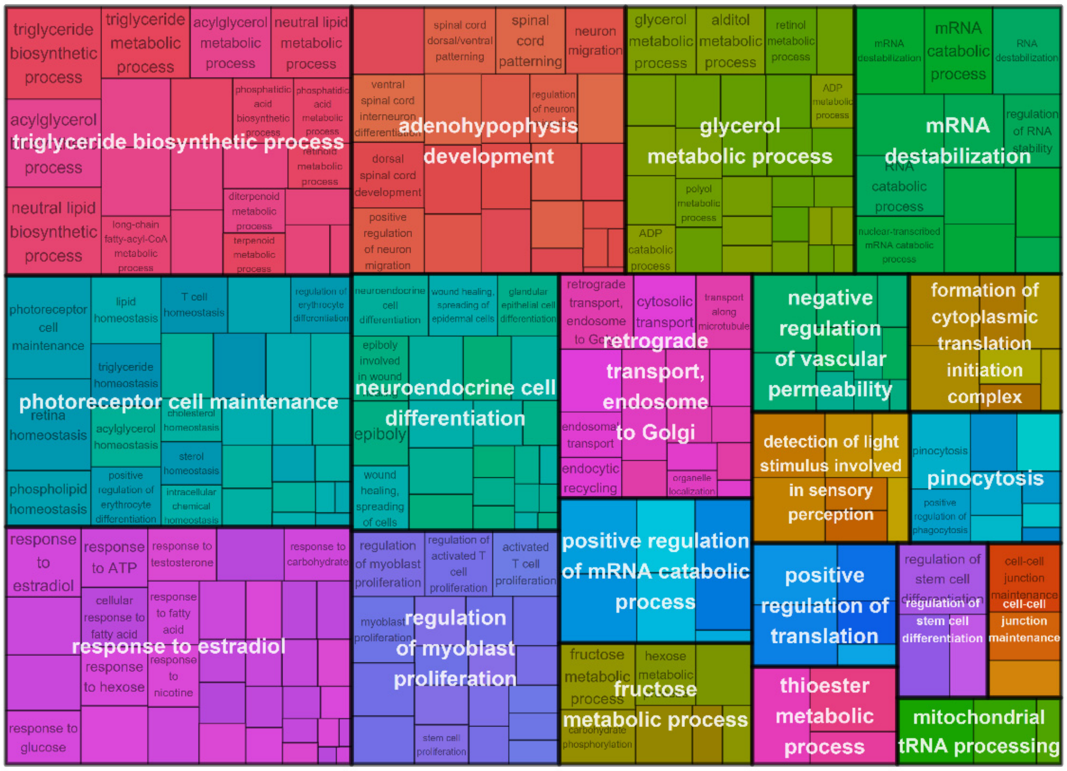

Supplement: Supplementary file 1 [file biology-15-00849-s001.zip › Supplementary Data5_TreeMap.pdf]
