# Supplementary material for: Circadian Transcriptomic Dynamics Identify Transferable Retina–Choroid Expression Patterns in Myopia Development via Multistage Machine Learning
Source: Biology (Basel). 2026 May 29;15(11):849. doi: 10.3390/biology15110849 (PMC13256023; doi:10.3390/biology15110849)

Supplementary Figure S1: Boruta feature-selection frequency across 50 repeated runs.

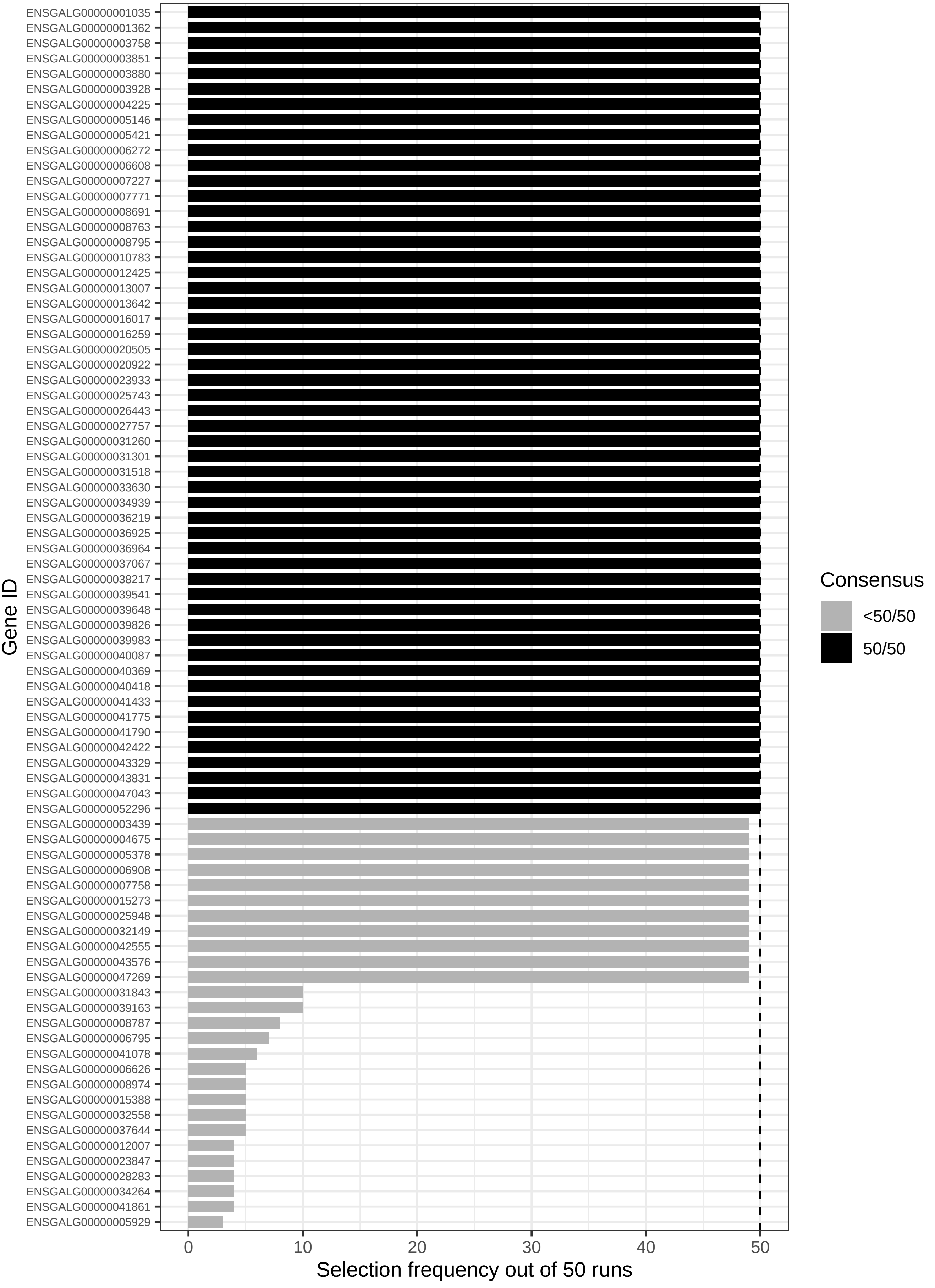

Supplementary Figure S2: SHAP global feature importance for the top 20 genes.

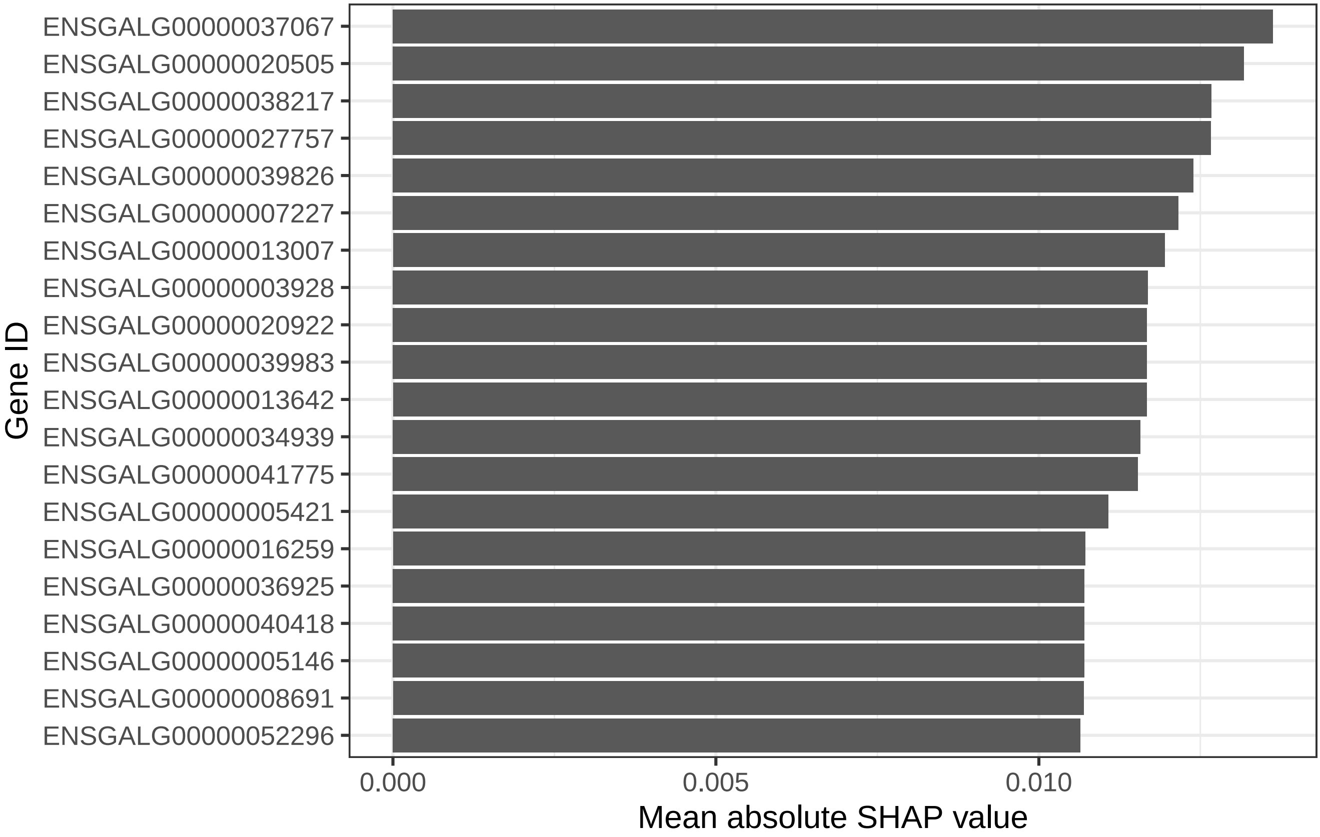

Supplementary Figure S3: SHAP summary plot for the top 20 Boruta genes.

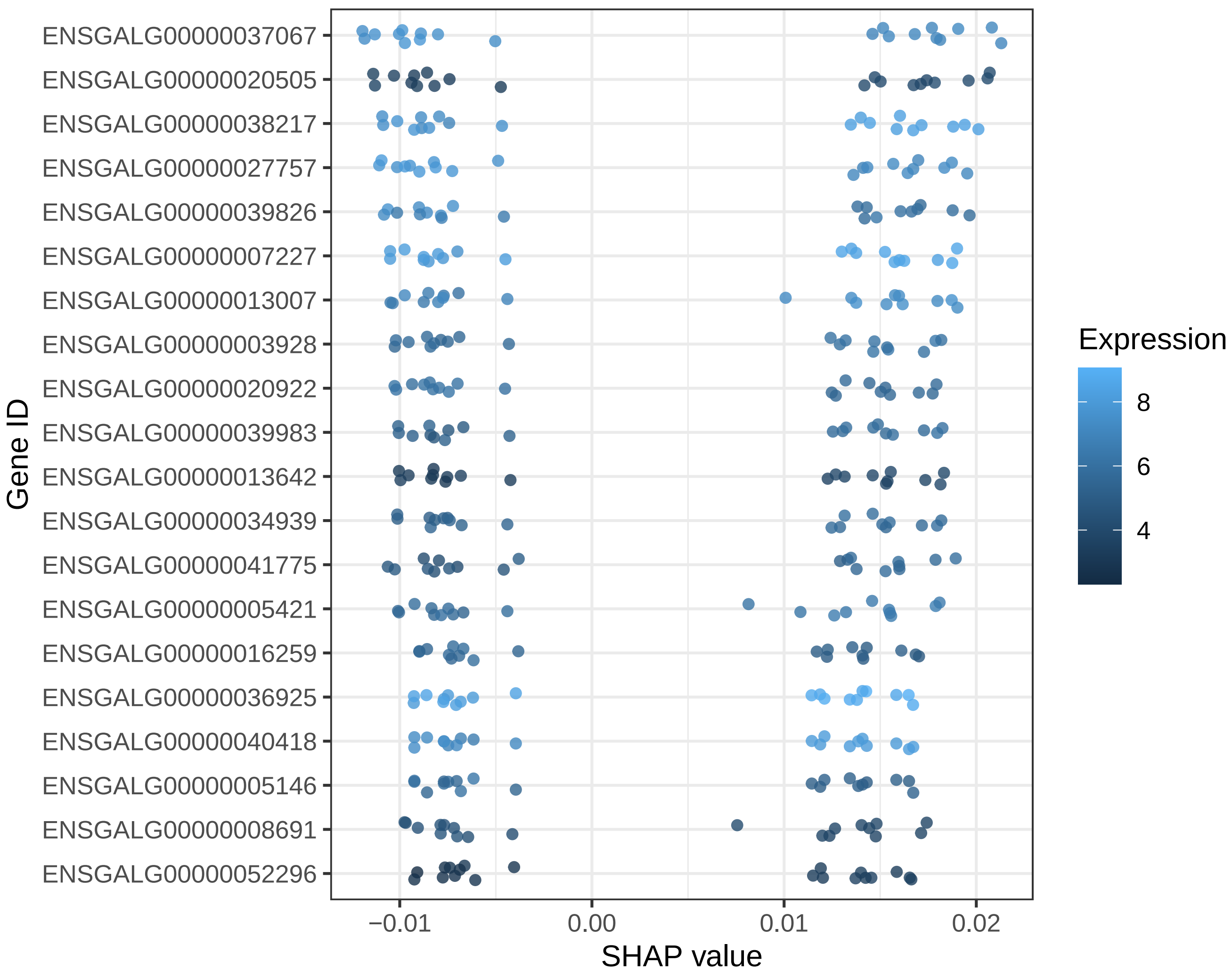

Supplement: Supplementary file 1 [file biology-15-00849-s001.zip › Supplementary Figures S1-3.pdf]
